# Supplementary material for: Clinical practice for migraine treatment and characteristics of medical facilities and physicians treating migraine: Insights from a retrospective cohort study using a Japanese claims database
Source: PLoS One. 2024 Dec 19;19(12):e0315610. doi: 10.1371/journal.pone.0315610 (PMC11658538; doi:10.1371/journal.pone.0315610)
Supplement: S2 Table — Subgroups were defined based on the initial diagnosis of migraine for the patients included in the migraine cohort. It is possible that the same patient may visit multiple facilities in the same month, and the total of the subgroups may not match the patient number of migraine cohort. Abbreviations: Anti-CGRP mAbs, anti-calcitonin gene-related peptide monoclonal antibodies; CP, clinic having ≤ 19-bed capacity; HP, hospital having ≥ 20-bed capacity; NSAIDs, nonsteroidal anti-inflammatory drugs. (DOCX) [file pone.0315610.s002.docx]

**S2 Table. Treatment for migraine for patients who initiated migraine treatment within the year**

| **Migraine cohort** | **2018–2023** | | **2018** | | **2019** | | | **2020** | | | **2021** | | | **2022** | | | **2023** | | |
| --- | --- | --- | --- | --- | --- | --- | --- | --- | --- | --- | --- | --- | --- | --- | --- | --- | --- | --- | --- |
|  | *N*=231,156 | - | *N*=39,714 | - | *N*=39,961 | - | *N*=38,425 | | - | *N*=47,071 | | - | *N*=44,140 | | - | *N*=21,845 | | - |  |
|  | *n* | % | *n* | % | *n* | % | *n* | | % | *n* | | % | *n* | | % | *n* | | % |  |
| Acute treatment | 219,666 | 95.0 | 37,997 | 95.7 | 38,056 | 95.2 | 36,575 | | 95.2 | 44,733 | | 95.0 | 41,699 | | 94.5 | 20,606 | | 94.3 |  |
| Triptan | 125,075 | 54.1 | 22,219 | 55.9 | 21,528 | 53.9 | 21,829 | | 56.8 | 25,066 | | 53.3 | 22,866 | | 51.8 | 11,567 | | 53.0 |  |
| Ergotamine | 5,808 | 2.5 | 1,343 | 3.4 | 1,158 | 2.9 | 1,081 | | 2.8 | 1,023 | | 2.2 | 797 | | 1.8 | 406 | | 1.9 |  |
| Acetaminophen and NSAIDs | 135,678 | 58.7 | 23,001 | 57.9 | 23,811 | 59.6 | 21,906 | | 57.0 | 28,245 | | 60.0 | 26,278 | | 59.5 | 12,437 | | 56.9 |  |
| Lasmiditan | 2,016 | 0.9 | 0 | 0.0 | 0 | 0.0 | 0 | | 0.0 | 0 | | 0.0 | 1,319 | | 3.0 | 697 | | 3.2 |  |
| Preventive treatment | 44,430 | 19.2 | 6,423 | 16.2 | 6,781 | 17.0 | 7,334 | | 19.1 | 9,342 | | 19.8 | 9,705 | | 22.0 | 4,845 | | 22.2 |  |
| Anti-CGRP mAbs | 488 | 0.2 | 0 | 0.0 | 0 | 0.0 | 0 | | 0.0 | 102 | | 0.2 | 259 | | 0.6 | 127 | | 0.6 |  |
| Antiepileptics | 14,245 | 6.2 | 2,235 | 5.6 | 2,299 | 5.8 | 2,512 | | 6.5 | 3,071 | | 6.5 | 2,775 | | 6.3 | 1,353 | | 6.2 |  |
| Antidepressants | 7,088 | 3.1 | 917 | 2.3 | 1,001 | 2.5 | 1,073 | | 2.8 | 1,454 | | 3.1 | 1,879 | | 4.3 | 764 | | 3.5 |  |
| Beta-blockers | 3,675 | 1.6 | 474 | 1.2 | 521 | 1.3 | 573 | | 1.5 | 710 | | 1.5 | 906 | | 2.1 | 491 | | 2.2 |  |
| Calcium channel blockers | 25,928 | 11.2 | 3,722 | 9.4 | 3,904 | 9.8 | 4,189 | | 10.9 | 5,539 | | 11.8 | 5,714 | | 12.9 | 2,860 | | 13.1 |  |
| Acute treatment only | 186,726 | 80.8 | 33,291 | 83.8 | 33,180 | 83.0 | 31,091 | | 80.9 | 37,729 | | 80.2 | 34,435 | | 78.0 | 17,000 | | 77.8 |  |
| Preventive treatment only | 11,490 | 5.0 | 1,717 | 4.3 | 1,905 | 4.8 | 1,850 | | 4.8 | 2,338 | | 5.0 | 2,441 | | 5.5 | 1,239 | | 5.7 |  |
| Acute and preventive treatment | 32,940 | 14.3 | 4,706 | 11.8 | 4,876 | 12.2 | 5,484 | | 14.3 | 7,004 | | 14.9 | 7,264 | | 16.5 | 3,606 | | 16.5 |  |
| **HP** | **2018–2023** | | **2018** | | **2019** | | | **2020** | | | **2021** | | | **2022** | | | **2023** | | |
|  | *N*=43,707 | 18.9 | *N*=7,673 | 19.3 | *N*=8,030 | 20.1 | *N*=7,661 | | 19.9 | *N*=8,626 | | 18.3 | *N*=7,957 | | 18.0 | *N*=3,760 | | 17.2 |  |
|  | *n* | % | *n* | % | *n* | % | *n* | | % | *n* | | % | *n* | | % | *n* | | % |  |
| Acute treatment | 41,790 | 95.6 | 7,365 | 96.0 | 7,706 | 96.0 | 7,352 | | 96.0 | 8,235 | | 95.5 | 7,575 | | 95.2 | 3,557 | | 94.6 |  |
| Triptan | 22,504 | 51.5 | 4,185 | 54.5 | 4,204 | 52.4 | 3,964 | | 51.7 | 4,311 | | 50.0 | 3,993 | | 50.2 | 1,847 | | 49.1 |  |
| Ergotamine | 473 | 1.1 | 89 | 1.2 | 102 | 1.3 | 112 | | 1.5 | 69 | | 0.8 | 73 | | 0.9 | 28 | | 0.7 |  |
| Acetaminophen and NSAIDs | 29,112 | 66.6 | 4,921 | 64.1 | 5,323 | 66.3 | 5,131 | | 67.0 | 5,885 | | 68.2 | 5,379 | | 67.6 | 2,473 | | 65.8 |  |
| Lasmiditan | 339 | 0.8 | 0 | 0.0 | 0 | 0.0 | 0 | | 0.0 | 0 | | 0.0 | 189 | | 2.4 | 150 | | 4.0 |  |
| Preventive treatment | 7,427 | 17.0 | 1,145 | 14.9 | 1,178 | 14.7 | 1,211 | | 15.8 | 1,466 | | 17.0 | 1,591 | | 20.0 | 836 | | 22.2 |  |
| Anti-CGRP mAbs | 184 | 0.4 | 0 | 0.0 | 0 | 0.0 | 0 | | 0.0 | 38 | | 0.4 | 103 | | 1.3 | 43 | | 1.1 |  |
| Antiepileptics | 2,281 | 5.2 | 391 | 5.1 | 398 | 5.0 | 404 | | 5.3 | 449 | | 5.2 | 434 | | 5.5 | 205 | | 5.5 |  |
| Antidepressants | 1,048 | 2.4 | 174 | 2.3 | 162 | 2.0 | 156 | | 2.0 | 192 | | 2.2 | 249 | | 3.1 | 115 | | 3.1 |  |
| Beta-blockers | 727 | 1.7 | 96 | 1.3 | 120 | 1.5 | 102 | | 1.3 | 125 | | 1.4 | 184 | | 2.3 | 100 | | 2.7 |  |
| Calcium channel blockers | 4,301 | 9.8 | 627 | 8.2 | 659 | 8.2 | 697 | | 9.1 | 897 | | 10.4 | 929 | | 11.7 | 492 | | 13.1 |  |
| Acute treatment only | 36,280 | 83.0 | 6,528 | 85.1 | 6,852 | 85.3 | 6,450 | | 84.2 | 7,160 | | 83.0 | 6,366 | | 80.0 | 2,924 | | 77.8 |  |
| Preventive treatment only | 1,917 | 4.4 | 308 | 4.0 | 324 | 4.0 | 309 | | 4.0 | 391 | | 4.5 | 382 | | 4.8 | 203 | | 5.4 |  |
| Acute and preventive treatment | 5,510 | 12.6 | 837 | 10.9 | 854 | 10.6 | 902 | | 11.8 | 1,075 | | 12.5 | 1,209 | | 15.2 | 633 | | 16.8 |  |
| **CP** | **2018–2023** | | **2018** | | **2019** | | | **2020** | | | **2021** | | | **2022** | | | **2023** | | |
|  | *N*=190,711 | 82.5 | *N*=32,679 | 82.3 | *N*=32,534 | 81.4 | *N*=31,337 | | 81.6 | *N*=39,077 | | 83.0 | *N*=36,786 | | 83.3 | *N*=18,298 | | 83.8 |  |
|  | *n* | % | *n* | % | *n* | % | *n* | | % | *n* | | % | *n* | | % | *n* | | % |  |
| Acute treatment | 180,833 | 94.8 | 31,220 | 95.5 | 30,905 | 95.0 | 29,746 | | 94.9 | 37,059 | | 94.8 | 34,667 | | 94.2 | 17,236 | | 94.2 |  |
| Triptan | 104,373 | 54.7 | 18,384 | 56.3 | 17,672 | 54.3 | 18,191 | | 58.0 | 21,120 | | 54.0 | 19,188 | | 52.2 | 9,818 | | 53.7 |  |
| Ergotamine | 5,348 | 2.8 | 1,256 | 3.8 | 1,059 | 3.3 | 972 | | 3.1 | 956 | | 2.4 | 726 | | 2.0 | 379 | | 2.1 |  |
| Acetaminophen and NSAIDs | 107,395 | 56.3 | 18,251 | 55.8 | 18,636 | 57.3 | 16,907 | | 54.0 | 22,516 | | 57.6 | 21,058 | | 57.2 | 10,027 | | 54.8 |  |
| Lasmiditan | 1,682 | 0.9 | 0 | 0.0 | 0 | 0.0 | 0 | | 0.0 | 0 | | 0.0 | 1,134 | | 3.1 | 548 | | 3.0 |  |
| Preventive treatment | 37,423 | 19.6 | 5,348 | 16.4 | 5,669 | 17.4 | 6,186 | | 19.7 | 7,965 | | 20.4 | 8,211 | | 22.3 | 4,044 | | 22.1 |  |
| Anti-CGRP mAbs | 307 | 0.2 | 0 | 0.0 | 0 | 0.0 | 0 | | 0.0 | 64 | | 0.2 | 159 | | 0.4 | 84 | | 0.5 |  |
| Antiepileptics | 12,051 | 6.3 | 1,858 | 5.7 | 1,913 | 5.9 | 2,124 | | 6.8 | 2,642 | | 6.8 | 2,360 | | 6.4 | 1,154 | | 6.3 |  |
| Antidepressants | 6,076 | 3.2 | 748 | 2.3 | 843 | 2.6 | 922 | | 2.9 | 1,272 | | 3.3 | 1,638 | | 4.5 | 653 | | 3.6 |  |
| Beta-blockers | 2,960 | 1.6 | 379 | 1.2 | 404 | 1.2 | 472 | | 1.5 | 587 | | 1.5 | 725 | | 2.0 | 393 | | 2.1 |  |
| Calcium channel blockers | 21,839 | 11.5 | 3,127 | 9.6 | 3,280 | 10.1 | 3,527 | | 11.3 | 4,688 | | 12.0 | 4,831 | | 13.1 | 2,386 | | 13.0 |  |
| Acute treatment only | 153,288 | 80.4 | 27,331 | 83.6 | 26,865 | 82.6 | 25,151 | | 80.3 | 31,112 | | 79.6 | 28,575 | | 77.7 | 14,254 | | 77.9 |  |
| Preventive treatment only | 9,878 | 5.2 | 1,459 | 4.5 | 1,629 | 5.0 | 1,591 | | 5.1 | 2,018 | | 5.2 | 2,119 | | 5.8 | 1,062 | | 5.8 |  |
| Acute and preventive treatment | 27,545 | 14.4 | 3,889 | 11.9 | 4,040 | 12.4 | 4,595 | | 14.7 | 5,947 | | 15.2 | 6,092 | | 16.6 | 2,982 | | 16.3 |  |
| **With specialist** | **2018–2023** | | **2018** | | **2019** | | | **2020** | | | **2021** | | | **2022** | | | **2023** | | |
|  | *N*=114,583 | 49.6 | *N*=20,233 | 50.9 | *N*=20,342 | 50.9 | *N*=19,967 | | 52.0 | *N*=23,088 | | 49.0 | *N*=20,894 | | 47.3 | *N*=10,059 | | 46.0 |  |
|  | *n* | % | *n* | % | *n* | % | *n* | | % | *n* | | % | *n* | | % | *n* | | % |  |
| Acute treatment | 108,537 | 94.7 | 19,289 | 95.3 | 19,255 | 94.7 | 18,976 | | 95.0 | 21,872 | | 94.7 | 19,659 | | 94.1 | 9,486 | | 94.3 |  |
| Triptan | 75,883 | 66.2 | 13,761 | 68.0 | 13,397 | 65.9 | 13,486 | | 67.5 | 15,269 | | 66.1 | 13,487 | | 64.5 | 6,483 | | 64.4 |  |
| Ergotamine | 1,804 | 1.6 | 383 | 1.9 | 348 | 1.7 | 372 | | 1.9 | 332 | | 1.4 | 242 | | 1.2 | 127 | | 1.3 |  |
| Acetaminophen and NSAIDs | 55,952 | 48.8 | 9,846 | 48.7 | 10,148 | 49.9 | 9,661 | | 48.4 | 11,329 | | 49.1 | 10,202 | | 48.8 | 4,766 | | 47.4 |  |
| Lasmiditan | 974 | 0.9 | 0 | 0.0 | 0 | 0.0 | 0 | | 0.0 | 0 | | 0.0 | 634 | | 3.0 | 340 | | 3.4 |  |
| Preventive treatment | 28,215 | 24.6 | 4,355 | 21.5 | 4,638 | 22.8 | 4,764 | | 23.9 | 5,904 | | 25.6 | 5,806 | | 27.8 | 2,748 | | 27.3 |  |
| Anti-CGRP mAbs | 344 | 0.3 | 0 | 0.0 | 0 | 0.0 | 0 | | 0.0 | 79 | | 0.3 | 191 | | 0.9 | 74 | | 0.7 |  |
| Antiepileptics | 8,785 | 7.7 | 1,460 | 7.2 | 1,508 | 7.4 | 1,583 | | 7.9 | 1,877 | | 8.1 | 1,642 | | 7.9 | 715 | | 7.1 |  |
| Antidepressants | 5,069 | 4.4 | 722 | 3.6 | 778 | 3.8 | 827 | | 4.1 | 1,084 | | 4.7 | 1,185 | | 5.7 | 473 | | 4.7 |  |
| Beta-blockers | 1,975 | 1.7 | 269 | 1.3 | 319 | 1.6 | 307 | | 1.5 | 388 | | 1.7 | 458 | | 2.2 | 234 | | 2.3 |  |
| Calcium channel blockers | 16,728 | 14.6 | 2,571 | 12.7 | 2,717 | 13.4 | 2,781 | | 13.9 | 3,532 | | 15.3 | 3,455 | | 16.5 | 1,672 | | 16.6 |  |
| Acute treatment only | 86,368 | 75.4 | 15,878 | 78.5 | 15,704 | 77.2 | 15,203 | | 76.1 | 17,184 | | 74.4 | 15,088 | | 72.2 | 7,311 | | 72.7 |  |
| Preventive treatment only | 6,046 | 5.3 | 944 | 4.7 | 1,087 | 5.3 | 991 | | 5.0 | 1,216 | | 5.3 | 1,235 | | 5.9 | 573 | | 5.7 |  |
| Acute and preventive treatment | 22,169 | 19.3 | 3,411 | 16.9 | 3,551 | 17.5 | 3,773 | | 18.9 | 4,688 | | 20.3 | 4,571 | | 21.9 | 2,175 | | 21.6 |  |
| **Without specialist** | **2018–2023** | | **2018** | | **2019** | | | **2020** | | | **2021** | | | **2022** | | | **2023** | | |
|  | *N*=121,008 | 52.3 | *N*=20,372 | 51.3 | *N*=20,431 | 51.1 | *N*=19,189 | | 49.9 | *N*=24,893 | | 52.9 | *N*=24,067 | | 54.5 | *N*=12,056 | | 55.2 |  |
|  | *n* | % | *n* | % | *n* | % | *n* | | % | *n* | | % | *n* | | % | *n* | | % |  |
| Acute treatment | 115,136 | 95.1 | 19,531 | 95.9 | 19,534 | 95.6 | 18,265 | | 95.2 | 23,675 | | 95.1 | 22,780 | | 94.7 | 11,351 | | 94.2 |  |
| Triptan | 51,791 | 42.8 | 9,000 | 44.2 | 8,619 | 42.2 | 8,788 | | 45.8 | 10,336 | | 41.5 | 9,831 | | 40.8 | 5,217 | | 43.3 |  |
| Ergotamine | 4,028 | 3.3 | 967 | 4.7 | 814 | 4.0 | 716 | | 3.7 | 693 | | 2.8 | 558 | | 2.3 | 280 | | 2.3 |  |
| Acetaminophen and NSAIDs | 80,701 | 66.7 | 13,356 | 65.6 | 13,830 | 67.7 | 12,408 | | 64.7 | 17,121 | | 68.8 | 16,247 | | 67.5 | 7,739 | | 64.2 |  |
| Lasmiditan | 1,052 | 0.9 | 0 | 0.0 | 0 | 0.0 | 0 | | 0.0 | 0 | | 0.0 | 693 | | 2.9 | 359 | | 3.0 |  |
| Preventive treatment | 16,728 | 13.8 | 2,136 | 10.5 | 2,228 | 10.9 | 2,644 | | 13.8 | 3,561 | | 14.3 | 4,015 | | 16.7 | 2,144 | | 17.8 |  |
| Anti-CGRP mAbs | 147 | 0.1 | 0 | 0.0 | 0 | 0.0 | 0 | | 0.0 | 23 | | 0.1 | 71 | | 0.3 | 53 | | 0.4 |  |
| Antiepileptics | 5,560 | 4.6 | 788 | 3.9 | 808 | 4.0 | 945 | | 4.9 | 1,222 | | 4.9 | 1,153 | | 4.8 | 644 | | 5.3 |  |
| Antidepressants | 2,055 | 1.7 | 200 | 1.0 | 230 | 1.1 | 251 | | 1.3 | 379 | | 1.5 | 702 | | 2.9 | 293 | | 2.4 |  |
| Beta-blockers | 1,715 | 1.4 | 207 | 1.0 | 204 | 1.0 | 268 | | 1.4 | 325 | | 1.3 | 451 | | 1.9 | 260 | | 2.2 |  |
| Calcium channel blockers | 9,474 | 7.8 | 1,185 | 5.8 | 1,239 | 6.1 | 1,448 | | 7.5 | 2,071 | | 8.3 | 2,316 | | 9.6 | 1,215 | | 10.1 |  |
| Acute treatment only | 104,280 | 86.2 | 18,236 | 89.5 | 18,203 | 89.1 | 16,545 | | 86.2 | 21,332 | | 85.7 | 20,052 | | 83.3 | 9,912 | | 82.2 |  |
| Preventive treatment only | 5,872 | 4.9 | 841 | 4.1 | 897 | 4.4 | 924 | | 4.8 | 1,218 | | 4.9 | 1,287 | | 5.3 | 705 | | 5.8 |  |
| Acute and preventive treatment | 10,856 | 9.0 | 1,295 | 6.4 | 1,331 | 6.5 | 1,720 | | 9.0 | 2,343 | | 9.4 | 2,728 | | 11.3 | 1,439 | | 11.9 |  |
| **HP with specialist** | **2018–2023** | | **2018** | | **2019** | | | **2020** | | | **2021** | | | **2022** | | | **2023** | | |
|  | *N*=36,470 | 15.8 | *N*=6,500 | 16.4 | *N*=6,756 | 16.9 | *N*=6,345 | | 16.5 | *N*=7,083 | | 15.0 | *N*=6,611 | | 15.0 | *N*=3,175 | | 14.5 |  |
|  | *n* | % | *n* | % | *n* | % | *n* | | % | *n* | | % | *n* | | % | *n* | | % |  |
| Acute treatment | 34,787 | 95.4 | 6,226 | 95.8 | 6,458 | 95.6 | 6,084 | | 95.9 | 6,745 | | 95.2 | 6,283 | | 95.0 | 2,991 | | 94.2 |  |
| Triptan | 20,057 | 55.0 | 3,763 | 57.9 | 3,756 | 55.6 | 3,526 | | 55.6 | 3,824 | | 54.0 | 3,555 | | 53.8 | 1,633 | | 51.4 |  |
| Ergotamine | 315 | 0.9 | 62 | 1.0 | 70 | 1.0 | 71 | | 1.1 | 49 | | 0.7 | 43 | | 0.7 | 20 | | 0.6 |  |
| Acetaminophen and NSAIDs | 23,507 | 64.5 | 4,048 | 62.3 | 4,346 | 64.3 | 4,119 | | 64.9 | 4,654 | | 65.7 | 4,324 | | 65.4 | 2,016 | | 63.5 |  |
| Lasmiditan | 314 | 0.9 | 0 | 0.0 | 0 | 0.0 | 0 | | 0.0 | 0 | | 0.0 | 174 | | 2.6 | 140 | | 4.4 |  |
| Preventive treatment | 6,700 | 18.4 | 1,037 | 16.0 | 1,086 | 16.1 | 1,067 | | 16.8 | 1,314 | | 18.6 | 1,421 | | 21.5 | 775 | | 24.4 |  |
| Anti-CGRP mAbs | 171 | 0.5 | 0 | 0.0 | 0 | 0.0 | 0 | | 0.0 | 35 | | 0.5 | 95 | | 1.4 | 41 | | 1.3 |  |
| Antiepileptics | 1,957 | 5.4 | 334 | 5.1 | 348 | 5.2 | 333 | | 5.2 | 381 | | 5.4 | 374 | | 5.7 | 187 | | 5.9 |  |
| Antidepressants | 980 | 2.7 | 169 | 2.6 | 155 | 2.3 | 145 | | 2.3 | 178 | | 2.5 | 224 | | 3.4 | 109 | | 3.4 |  |
| Beta-blockers | 626 | 1.7 | 87 | 1.3 | 107 | 1.6 | 84 | | 1.3 | 103 | | 1.5 | 157 | | 2.4 | 88 | | 2.8 |  |
| Calcium channel blockers | 3,993 | 10.9 | 584 | 9.0 | 626 | 9.3 | 639 | | 10.1 | 831 | | 11.7 | 850 | | 12.9 | 463 | | 14.6 |  |
| Acute treatment only | 29,770 | 81.6 | 5,463 | 84.0 | 5,670 | 83.9 | 5,278 | | 83.2 | 5,769 | | 81.4 | 5,190 | | 78.5 | 2,400 | | 75.6 |  |
| Preventive treatment only | 1,683 | 4.6 | 274 | 4.2 | 298 | 4.4 | 261 | | 4.1 | 338 | | 4.8 | 328 | | 5.0 | 184 | | 5.8 |  |
| Acute and preventive treatment | 5,017 | 13.8 | 763 | 11.7 | 788 | 11.7 | 806 | | 12.7 | 976 | | 13.8 | 1,093 | | 16.5 | 591 | | 18.6 |  |
| **HP without specialist** | **2018–2023** | | **2018** | | **2019** | | | **2020** | | | **2021** | | | **2022** | | | **2023** | | |
|  | *N*=7,372 | 3.2 | *N*=1,196 | 3.0 | *N*=1,301 | 3.3 | *N*=1,338 | | 3.5 | *N*=1,576 | | 3.3 | *N*=1,367 | | 3.1 | *N*=594 | | 2.7 |  |
|  | *n* | % | *n* | % | *n* | % | *n* | | % | *n* | | % | *n* | | % | *n* | | % |  |
| Acute treatment | 7,129 | 96.7 | 1,160 | 97.0 | 1,273 | 97.8 | 1,289 | | 96.3 | 1,522 | | 96.6 | 1,311 | | 95.9 | 574 | | 96.6 |  |
| Triptan | 2,511 | 34.1 | 434 | 36.3 | 463 | 35.6 | 446 | | 33.3 | 507 | | 32.2 | 445 | | 32.6 | 216 | | 36.4 |  |
| Ergotamine | 158 | 2.1 | 27 | 2.3 | 32 | 2.5 | 41 | | 3.1 | 20 | | 1.3 | 30 | | 2.2 | 8 | | 1.3 |  |
| Acetaminophen and NSAIDs | 5,663 | 76.8 | 882 | 73.7 | 987 | 75.9 | 1,022 | | 76.4 | 1,246 | | 79.1 | 1,066 | | 78.0 | 460 | | 77.4 |  |
| Lasmiditan | 25 | 0.3 | 0 | 0.0 | 0 | 0.0 | 0 | | 0.0 | 0 | | 0.0 | 15 | | 1.1 | 10 | | 1.7 |  |
| Preventive treatment | 742 | 10.1 | 110 | 9.2 | 95 | 7.3 | 147 | | 11.0 | 154 | | 9.8 | 174 | | 12.7 | 62 | | 10.4 |  |
| Anti-CGRP mAbs | 13 | 0.2 | 0 | 0.0 | 0 | 0.0 | 0 | | 0.0 | 3 | | 0.2 | 8 | | 0.6 | 2 | | 0.3 |  |
| Antiepileptics | 327 | 4.4 | 58 | 4.8 | 50 | 3.8 | 72 | | 5.4 | 68 | | 4.3 | 61 | | 4.5 | 18 | | 3.0 |  |
| Antidepressants | 68 | 0.9 | 5 | 0.4 | 7 | 0.5 | 11 | | 0.8 | 14 | | 0.9 | 25 | | 1.8 | 6 | | 1.0 |  |
| Beta-blockers | 101 | 1.4 | 9 | 0.8 | 13 | 1.0 | 18 | | 1.3 | 22 | | 1.4 | 27 | | 2.0 | 12 | | 2.0 |  |
| Calcium channel blockers | 319 | 4.3 | 44 | 3.7 | 35 | 2.7 | 60 | | 4.5 | 68 | | 4.3 | 82 | | 6.0 | 30 | | 5.1 |  |
| Acute treatment only | 6,630 | 89.9 | 1,086 | 90.8 | 1,206 | 92.7 | 1,191 | | 89.0 | 1,422 | | 90.2 | 1,193 | | 87.3 | 532 | | 89.6 |  |
| Preventive treatment only | 243 | 3.3 | 36 | 3.0 | 28 | 2.2 | 49 | | 3.7 | 54 | | 3.4 | 56 | | 4.1 | 20 | | 3.4 |  |
| Acute and preventive treatment | 499 | 6.8 | 74 | 6.2 | 67 | 5.1 | 98 | | 7.3 | 100 | | 6.3 | 118 | | 8.6 | 42 | | 7.1 |  |
| **CP with specialist** | **2018–2023** | | **2018** | | **2019** | | | **2020** | | | **2021** | | | **2022** | | | **2023** | | |
|  | *N*=79,559 | 34.4 | *N*=14,025 | 35.3 | *N*=13,883 | 34.7 | *N*=13,881 | | 36.1 | *N*=16,280 | | 34.6 | *N*=14,522 | | 32.9 | *N*=6,968 | | 31.9 |  |
|  | *n* | % | *n* | % | *n* | % | *n* | | % | *n* | | % | *n* | | % | *n* | | % |  |
| Acute treatment | 75,052 | 94.3 | 13,329 | 95.0 | 13,074 | 94.2 | 13,124 | | 94.5 | 15,369 | | 94.4 | 13,590 | | 93.6 | 6,566 | | 94.2 |  |
| Triptan | 56,662 | 71.2 | 10,172 | 72.5 | 9,824 | 70.8 | 10,105 | | 72.8 | 11,611 | | 71.3 | 10,060 | | 69.3 | 4,890 | | 70.2 |  |
| Ergotamine | 1,490 | 1.9 | 321 | 2.3 | 278 | 2.0 | 301 | | 2.2 | 284 | | 1.7 | 199 | | 1.4 | 107 | | 1.5 |  |
| Acetaminophen and NSAIDs | 32,763 | 41.2 | 5,869 | 41.8 | 5,869 | 42.3 | 5,594 | | 40.3 | 6,734 | | 41.4 | 5,932 | | 40.8 | 2,765 | | 39.7 |  |
| Lasmiditan | 661 | 0.8 | 0 | 0.0 | 0 | 0.0 | 0 | | 0.0 | 0 | | 0.0 | 461 | | 3.2 | 200 | | 2.9 |  |
| Preventive treatment | 21,724 | 27.3 | 3,357 | 23.9 | 3,583 | 25.8 | 3,723 | | 26.8 | 4,640 | | 28.5 | 4,428 | | 30.5 | 1,993 | | 28.6 |  |
| Anti-CGRP mAbs | 174 | 0.2 | 0 | 0.0 | 0 | 0.0 | 0 | | 0.0 | 44 | | 0.3 | 97 | | 0.7 | 33 | | 0.5 |  |
| Antiepileptics | 6,882 | 8.7 | 1,138 | 8.1 | 1,168 | 8.4 | 1,255 | | 9.0 | 1,511 | | 9.3 | 1,277 | | 8.8 | 533 | | 7.6 |  |
| Antidepressants | 4,112 | 5.2 | 556 | 4.0 | 624 | 4.5 | 685 | | 4.9 | 913 | | 5.6 | 966 | | 6.7 | 368 | | 5.3 |  |
| Beta-blockers | 1,355 | 1.7 | 183 | 1.3 | 213 | 1.5 | 224 | | 1.6 | 286 | | 1.8 | 302 | | 2.1 | 147 | | 2.1 |  |
| Calcium channel blockers | 12,819 | 16.1 | 1,998 | 14.2 | 2,104 | 15.2 | 2,155 | | 15.5 | 2,721 | | 16.7 | 2,624 | | 18.1 | 1,217 | | 17.5 |  |
| Acute treatment only | 57,835 | 72.7 | 10,668 | 76.1 | 10,300 | 74.2 | 10,158 | | 73.2 | 11,640 | | 71.5 | 10,094 | | 69.5 | 4,975 | | 71.4 |  |
| Preventive treatment only | 4,507 | 5.7 | 696 | 5.0 | 809 | 5.8 | 757 | | 5.5 | 911 | | 5.6 | 932 | | 6.4 | 402 | | 5.8 |  |
| Acute and preventive treatment | 17,217 | 21.6 | 2,661 | 19.0 | 2,774 | 20.0 | 2,966 | | 21.4 | 3,729 | | 22.9 | 3,496 | | 24.1 | 1,591 | | 22.8 |  |
| **CP without specialist** | **2018–2023** | | **2018** | | **2019** | | | **2020** | | | **2021** | | | **2022** | | | **2023** | | |
|  | *N*=113,818 | 49.2 | *N*=19,208 | 48.4 | *N*=19,154 | 47.9 | *N*=17,887 | | 46.6 | *N*=23,352 | | 49.6 | *N*=22,740 | | 51.5 | *N*=11,477 | | 52.5 |  |
|  | *n* | % | *n* | % | *n* | % | *n* | | % | *n* | | % | *n* | | % | *n* | | % |  |
| Acute treatment | 108,169 | 95.0 | 18,402 | 95.8 | 18,283 | 95.5 | 17,010 | | 95.1 | 22,181 | | 95.0 | 21,503 | | 94.6 | 10,790 | | 94.0 |  |
| Triptan | 49,369 | 43.4 | 8,581 | 44.7 | 8,169 | 42.6 | 8,362 | | 46.7 | 9,846 | | 42.2 | 9,402 | | 41.3 | 5,009 | | 43.6 |  |
| Ergotamine | 3,871 | 3.4 | 940 | 4.9 | 782 | 4.1 | 675 | | 3.8 | 673 | | 2.9 | 529 | | 2.3 | 272 | | 2.4 |  |
| Acetaminophen and NSAIDs | 75,090 | 66.0 | 12,483 | 65.0 | 12,849 | 67.1 | 11,394 | | 63.7 | 15,886 | | 68.0 | 15,195 | | 66.8 | 7,283 | | 63.5 |  |
| Lasmiditan | 1,027 | 0.9 | 0 | 0.0 | 0 | 0.0 | 0 | | 0.0 | 0 | | 0.0 | 678 | | 3.0 | 349 | | 3.0 |  |
| Preventive treatment | 16,005 | 14.1 | 2,026 | 10.5 | 2,134 | 11.1 | 2,502 | | 14.0 | 3,412 | | 14.6 | 3,847 | | 16.9 | 2,084 | | 18.2 |  |
| Anti-CGRP mAbs | 134 | 0.1 | 0 | 0.0 | 0 | 0.0 | 0 | | 0.0 | 20 | | 0.1 | 63 | | 0.3 | 51 | | 0.4 |  |
| Antiepileptics | 5,239 | 4.6 | 730 | 3.8 | 758 | 4.0 | 876 | | 4.9 | 1,155 | | 4.9 | 1,094 | | 4.8 | 626 | | 5.5 |  |
| Antidepressants | 1,988 | 1.7 | 195 | 1.0 | 223 | 1.2 | 240 | | 1.3 | 365 | | 1.6 | 678 | | 3.0 | 287 | | 2.5 |  |
| Beta-blockers | 1,614 | 1.4 | 198 | 1.0 | 191 | 1.0 | 250 | | 1.4 | 303 | | 1.3 | 424 | | 1.9 | 248 | | 2.2 |  |
| Calcium channel blockers | 9,166 | 8.1 | 1,141 | 5.9 | 1,205 | 6.3 | 1,390 | | 7.8 | 2,007 | | 8.6 | 2,236 | | 9.8 | 1,187 | | 10.3 |  |
| Acute treatment only | 97,813 | 85.9 | 17,182 | 89.5 | 17,020 | 88.9 | 15,385 | | 86.0 | 19,940 | | 85.4 | 18,893 | | 83.1 | 9,393 | | 81.8 |  |
| Preventive treatment only | 5,649 | 5.0 | 806 | 4.2 | 871 | 4.5 | 877 | | 4.9 | 1,171 | | 5.0 | 1,237 | | 5.4 | 687 | | 6.0 |  |
| Acute and preventive treatment | 10,356 | 9.1 | 1,220 | 6.4 | 1,263 | 6.6 | 1,625 | | 9.1 | 2,241 | | 9.6 | 2,610 | | 11.5 | 1,397 | | 12.2 |  |

Subgroups were defined based on the initial diagnosis of migraine for the patients included in the migraine cohort. It is possible that the same patient may visit multiple facilities in the same month, and the total of the subgroups may not match the patient number of migraine cohort.

*Abbreviations: Anti-CGRP mAbs*, anti-calcitonin gene-related peptide monoclonal antibodies; *CP*, clinic having ≤ 19-bed capacity; *HP*, hospital having ≥ 20-bed capacity; *NSAID*s, nonsteroidal anti-inflammatory drugs.
