# Supplementary material for: Clinical practice for migraine treatment and characteristics of medical facilities and physicians treating migraine: Insights from a retrospective cohort study using a Japanese claims database
Source: PLoS One. 2024 Dec 19;19(12):e0315610. doi: 10.1371/journal.pone.0315610 (PMC11658538; doi:10.1371/journal.pone.0315610)
Supplement: S3 Table — If >1 class of treatment started on the same day, each class was counted. Subgroups were defined based on the initial diagnosis of migraine for the patients included in the migraine cohort. It is possible that the same patient may visit multiple facilities in the same month, and the total of the subgroups may not match the patient number of migraine cohort. Abbreviations: Anti-CGRP mAbs, anti-calcitonin gene-related peptide monoclonal antibodies; CP, clinic having ≤ 19-bed capacity; HP, hospital having ≥ 20-bed capacity; NSAIDs, non-steroidal anti-inflammatory drugs. (DOCX) [file pone.0315610.s003.docx]

**S3 Table. Treatment prescriptions in migraine cohort and subgroups**

| **Total migraine cohort** | **First prescription** | | **Second prescription** | | **Third prescription** | | **Fourth prescription** | |
| --- | --- | --- | --- | --- | --- | --- | --- | --- |
|  | *N*=231,156 | - | *N*=40,360 | - | *N*=8,565 | - | *N*=2,478 | - |
|  | *n* | % | *n* | % | *n* | % | *n* | % |
| Any acute treatment | 217,573 | 94.1 | 28,246 | 70.0 | 3,314 | 38.7 | 737 | 29.7 |
| Acetaminophen and/or NSAIDs | 126,398 | 54.7 | 16,463 | 40.8 | 1,726 | 20.2 | 296 | 11.9 |
| Triptan | 86,654 | 37.5 | 9,820 | 24.3 | 711 | 8.3 | 79 | 3.2 |
| Ergotamine | 3,696 | 1.6 | 870 | 2.2 | 176 | 2.1 | 45 | 1.8 |
| Lasmiditan | 825 | 0.4 | 1,093 | 2.7 | 701 | 8.2 | 317 | 12.8 |
| Any preventive treatment | 37,365 | 16.2 | 14,408 | 35.7 | 5,509 | 64.3 | 1,787 | 72.1 |
| Anti-CGRP mAbs | 117 | 0.1 | 358 | 0.9 | 486 | 5.7 | 388 | 15.7 |
| Antiepileptics | 10,947 | 4.7 | 4,185 | 10.4 | 1,630 | 19.0 | 478 | 19.3 |
| Antidepressants | 4,997 | 2.2 | 2,523 | 6.3 | 1,163 | 13.6 | 412 | 16.6 |
| Beta-blockers | 2,746 | 1.2 | 1,083 | 2.7 | 589 | 6.9 | 232 | 9.4 |
| Calcium channel blockers | 21,180 | 9.2 | 6,899 | 17.1 | 1,797 | 21.0 | 310 | 12.5 |
| **HP** | **First prescription** | | **Second prescription** | | **Third prescription** | | **Fourth prescription** | |
|  | *N*=42,102 | 18.2 | *N*=8,918 | 22.1 | *N*=1,973 | 23.0 | *N*=608 | 24.5 |
|  | *n* | % | *n* | % | *n* | % | *n* | % |
| Any acute treatment | 39,849 | 94.6 | 6,571 | 73.7 | 718 | 36.4 | 179 | 29.4 |
| Acetaminophen and/or NSAIDs | 26,468 | 62.9 | 3,740 | 41.9 | 374 | 19.0 | 78 | 12.8 |
| Triptan | 12,993 | 30.9 | 2,564 | 28.8 | 185 | 9.4 | 29 | 4.8 |
| Ergotamine | 272 | 0.6 | 89 | 1.0 | 18 | 0.9 | 2 | 0.3 |
| Lasmiditan | 116 | 0.3 | 178 | 2.0 | 141 | 7.1 | 70 | 11.5 |
| Any preventive treatment | 5,432 | 12.9 | 2,895 | 32.5 | 1,330 | 67.4 | 439 | 72.2 |
| Anti-CGRP mAbs | 45 | 0.1 | 131 | 1.5 | 166 | 8.4 | 118 | 19.4 |
| Antiepileptics | 1,549 | 3.7 | 754 | 8.5 | 350 | 17.7 | 104 | 17.1 |
| Antidepressants | 631 | 1.5 | 422 | 4.7 | 239 | 12.1 | 109 | 17.9 |
| Beta-blockers | 469 | 1.1 | 258 | 2.9 | 166 | 8.4 | 42 | 6.9 |
| Calcium channel blockers | 3,039 | 7.2 | 1,441 | 16.2 | 433 | 21.9 | 72 | 11.8 |
| **CP** | **First prescription** | | **Second prescription** | | **Third prescription** | | **Fourth prescription** | |
|  | *N*=189,124 | 81.8 | *N*=31,455 | 77.9 | *N*=6,593 | 77.0 | *N*=1,870 | 75.5 |
|  | *n* | % | *n* | % | *n* | % | *n* | % |
| Any acute treatment | 177,794 | 94.0 | 21,688 | 68.9 | 2,597 | 39.4 | 558 | 29.8 |
| Acetaminophen and/or NSAIDs | 99,985 | 52.9 | 12,725 | 40.5 | 1,353 | 20.5 | 218 | 11.7 |
| Triptan | 73,676 | 39.0 | 7,267 | 23.1 | 526 | 8.0 | 50 | 2.7 |
| Ergotamine | 3,424 | 1.8 | 781 | 2.5 | 158 | 2.4 | 43 | 2.3 |
| Lasmiditan | 709 | 0.4 | 915 | 2.9 | 560 | 8.5 | 247 | 13.2 |
| Any preventive treatment | 31,947 | 16.9 | 11,519 | 36.6 | 4,179 | 63.4 | 1,348 | 72.1 |
| Anti-CGRP mAbs | 72 | 0.0 | 227 | 0.7 | 320 | 4.9 | 270 | 14.4 |
| Antiepileptics | 9,401 | 5.0 | 3,432 | 10.9 | 1,280 | 19.4 | 374 | 20.0 |
| Antidepressants | 4,368 | 2.3 | 2,104 | 6.7 | 924 | 14.0 | 303 | 16.2 |
| Beta-blockers | 2,277 | 1.2 | 825 | 2.6 | 423 | 6.4 | 190 | 10.2 |
| Calcium channel blockers | 18,151 | 9.6 | 5,461 | 17.4 | 1,364 | 20.7 | 238 | 12.7 |
| **With specialists** | **First prescription** | | **Second prescription** | | **Third prescription** | | **Fourth prescription** | |
|  | *N*=112,332 | 48.6 | *N*=22,789 | 56.5 | *N*=5,426 | 63.4 | *N*=1,675 | 67.6 |
|  | *n* | % | *n* | % | *n* | % | *n* | % |
| Any acute treatment | 105,288 | 93.7 | 14,906 | 65.4 | 1,898 | 35.0 | 455 | 27.2 |
| Acetaminophen and/or NSAIDs | 50,477 | 44.9 | 8,454 | 37.1 | 987 | 18.2 | 191 | 11.4 |
| Triptan | 53,387 | 47.5 | 5,546 | 24.3 | 428 | 7.9 | 57 | 3.4 |
| Ergotamine | 1,056 | 0.9 | 304 | 1.3 | 69 | 1.3 | 20 | 1.2 |
| Lasmiditan | 368 | 0.3 | 602 | 2.6 | 414 | 7.6 | 187 | 11.2 |
| Any preventive treatment | 23,235 | 20.7 | 9,427 | 41.4 | 3,708 | 68.3 | 1,253 | 74.8 |
| Anti-CGRP mAbs | 66 | 0.1 | 274 | 1.2 | 390 | 7.2 | 294 | 17.6 |
| Antiepileptics | 6,551 | 5.8 | 2,723 | 11.9 | 1,098 | 20.2 | 321 | 19.2 |
| Antidepressants | 3,611 | 3.2 | 1,712 | 7.5 | 784 | 14.4 | 295 | 17.6 |
| Beta-blockers | 1,357 | 1.2 | 686 | 3.0 | 406 | 7.5 | 161 | 9.6 |
| Calcium channel blockers | 13,463 | 12.0 | 4,483 | 19.7 | 1,145 | 21.1 | 209 | 12.5 |
| **Without specialists** | **First prescription** | | **Second prescription** | | **Third prescription** | | **Fourth prescription** | |
|  | *N*=118,897 | 51.4 | *N*=17,586 | 43.6 | *N*=3,140 | 36.7 | *N*=803 | 32.4 |
|  | *n* | % | *n* | % | *n* | % | *n* | % |
| Any acute treatment | 112,358 | 94.5 | 13,355 | 75.9 | 1,417 | 45.1 | 282 | 35.1 |
| Acetaminophen and/or NSAIDs | 75,976 | 63.9 | 8,013 | 45.6 | 740 | 23.6 | 105 | 13.1 |
| Triptan | 33,284 | 28.0 | 4,284 | 24.4 | 283 | 9.0 | 22 | 2.7 |
| Ergotamine | 2,640 | 2.2 | 567 | 3.2 | 107 | 3.4 | 25 | 3.1 |
| Lasmiditan | 458 | 0.4 | 491 | 2.8 | 287 | 9.1 | 130 | 16.2 |
| Any preventive treatment | 14,146 | 11.9 | 4,991 | 28.4 | 1,801 | 57.4 | 534 | 66.5 |
| Anti-CGRP mAbs | 51 | 0.0 | 84 | 0.5 | 96 | 3.1 | 94 | 11.7 |
| Antiepileptics | 4,402 | 3.7 | 1,464 | 8.3 | 532 | 16.9 | 157 | 19.6 |
| Antidepressants | 1,388 | 1.2 | 813 | 4.6 | 379 | 12.1 | 117 | 14.6 |
| Beta-blockers | 1,392 | 1.2 | 397 | 2.3 | 183 | 5.8 | 71 | 8.8 |
| Calcium channel blockers | 7,726 | 6.5 | 2,422 | 13.8 | 652 | 20.8 | 101 | 12.6 |
| **HP with specialist** | **First prescription** | | **Second prescription** | | **Third prescription** | | **Fourth prescription** | |
|  | *N*=34,964 | 15.1 | *N*=7,903 | 19.6 | *N*=1,817 | 21.2 | *N*=571 | 23.0 |
|  | *n* | % | *n* | % | *n* | % | *n* | % |
| Any acute treatment | 32,995 | 94.4 | 5,749 | 72.7 | 650 | 35.8 | 166 | 29.1 |
| Acetaminophen and/or NSAIDs | 21,181 | 60.6 | 3,216 | 40.7 | 337 | 18.5 | 72 | 12.6 |
| Triptan | 11,532 | 33.0 | 2,304 | 29.2 | 171 | 9.4 | 26 | 4.6 |
| Ergotamine | 176 | 0.5 | 64 | 0.8 | 13 | 0.7 | 1 | 0.2 |
| Lasmiditan | 106 | 0.3 | 165 | 2.1 | 129 | 7.1 | 67 | 11.7 |
| Any preventive treatment | 4,855 | 13.9 | 2,667 | 33.7 | 1,235 | 68.0 | 415 | 72.7 |
| Anti-CGRP mAbs | 43 | 0.1 | 121 | 1.5 | 160 | 8.8 | 109 | 19.1 |
| Antiepileptics | 1,299 | 3.7 | 666 | 8.4 | 319 | 17.6 | 101 | 17.7 |
| Antidepressants | 591 | 1.7 | 395 | 5.0 | 221 | 12.2 | 104 | 18.2 |
| Beta-blockers | 390 | 1.1 | 232 | 2.9 | 155 | 8.5 | 40 | 7.0 |
| Calcium channel blockers | 2,806 | 8.0 | 1,356 | 17.2 | 404 | 22.2 | 67 | 11.7 |
| **HP without specialist** | **First prescription** | | **Second prescription** | | **Third prescription** | | **Fourth prescription** | |
|  | *N*=7,140 | 3.1 | *N*=1,015 | 2.5 | *N*=156 | 1.8 | *N*=37 | 1.5 |
|  | *n* | % | *n* | % | *n* | % | *n* | % |
| Any acute treatment | 6,856 | 96.0 | 822 | 81.0 | 68 | 43.6 | 13 | 35.1 |
| Acetaminophen and/or NSAIDs | 5,289 | 74.1 | 524 | 51.6 | 37 | 23.7 | 6 | 16.2 |
| Triptan | 1,461 | 20.5 | 260 | 25.6 | 14 | 9.0 | 3 | 8.1 |
| Ergotamine | 96 | 1.3 | 25 | 2.5 | 5 | 3.2 | 1 | 2.7 |
| Lasmiditan | 10 | 0.1 | 13 | 1.3 | 12 | 7.7 | 3 | 8.1 |
| Any preventive treatment | 578 | 8.1 | 228 | 22.5 | 95 | 60.9 | 24 | 64.9 |
| Anti-CGRP mAbs | 2 | 0.0 | 10 | 1.0 | 6 | 3.8 | 9 | 24.3 |
| Antiepileptics | 251 | 3.5 | 88 | 8.7 | 31 | 19.9 | 3 | 8.1 |
| Antidepressants | 40 | 0.6 | 27 | 2.7 | 18 | 11.5 | 5 | 13.5 |
| Beta-blockers | 80 | 1.1 | 26 | 2.6 | 11 | 7.1 | 2 | 5.4 |
| Calcium channel blockers | 233 | 3.3 | 85 | 8.4 | 29 | 18.6 | 5 | 13.5 |
| **CP with specialists** | **First prescription** | | **Second prescription** | | **Third prescription** | | **Fourth prescription** | |
|  | *N*=77,402 | 33.5 | *N*=14,891 | *N*=36.9 | *N*=3,609 | 42.1 | *N*=1,104 | 44.6 |
|  | *n* | % | *n* | % | *n* | % | *n* | % |
| Any acute treatment | 72,327 | 93.4 | 9,162 | 61.5 | 1,248 | 34.6 | 289 | 26.2 |
| Acetaminophen and/or NSAIDs | 29,319 | 37.9 | 5,239 | 35.2 | 650 | 18.0 | 119 | 10.8 |
| Triptan | 41,866 | 54.1 | 3,246 | 21.8 | 257 | 7.1 | 31 | 2.8 |
| Ergotamine | 880 | 1.1 | 240 | 1.6 | 56 | 1.6 | 19 | 1.7 |
| Lasmiditan | 262 | 0.3 | 437 | 2.9 | 285 | 7.9 | 120 | 10.9 |
| Any preventive treatment | 18,387 | 23.8 | 6,762 | 45.4 | 2,473 | 68.5 | 838 | 75.9 |
| Anti-CGRP mAbs | 23 | 0.0 | 153 | 1.0 | 230 | 6.4 | 185 | 16.8 |
| Antiepileptics | 5,254 | 6.8 | 2,058 | 13.8 | 779 | 21.6 | 220 | 19.9 |
| Antidepressants | 3,021 | 3.9 | 1,318 | 8.9 | 563 | 15.6 | 191 | 17.3 |
| Beta-blockers | 967 | 1.2 | 454 | 3.0 | 251 | 7.0 | 121 | 11.0 |
| Calcium channel blockers | 10,661 | 13.8 | 3,128 | 21.0 | 741 | 20.5 | 142 | 12.9 |
| **CP without specialists** | **First prescription** | | **Second prescription** | | **Third prescription** | | **Fourth prescription** | |
|  | *N*=111,758 | 48.3 | *N*=16,571 | 41.1 | *N*=2,984 | 34.8 | *N*=766 | 30.9 |
|  | *n* | % | *n* | % | *n* | % | *n* | % |
| Any acute treatment | 105,503 | 94.4 | 12,533 | 75.6 | 1,349 | 45.2 | 269 | 35.1 |
| Acetaminophen and/or NSAIDs | 70,688 | 63.3 | 7,489 | 45.2 | 703 | 23.6 | 99 | 12.9 |
| Triptan | 31,823 | 28.5 | 4,024 | 24.3 | 269 | 9.0 | 19 | 2.5 |
| Ergotamine | 2,544 | 2.3 | 542 | 3.3 | 102 | 3.4 | 24 | 3.1 |
| Lasmiditan | 448 | 0.4 | 478 | 2.9 | 275 | 9.2 | 127 | 16.6 |
| Any preventive treatment | 13,570 | 12.1 | 4,763 | 28.7 | 1,706 | 57.2 | 510 | 66.6 |
| Anti-CGRP mAbs | 49 | 0.0 | 74 | 0.4 | 90 | 3.0 | 85 | 11.1 |
| Antiepileptics | 4,151 | 3.7 | 1,376 | 8.3 | 501 | 16.8 | 154 | 20.1 |
| Antidepressants | 1,348 | 1.2 | 786 | 4.7 | 361 | 12.1 | 112 | 14.6 |
| Beta-blockers | 1,312 | 1.2 | 371 | 2.2 | 172 | 5.8 | 69 | 9.0 |
| Calcium channel blockers | 7,495 | 6.7 | 2,337 | 14.1 | 623 | 20.9 | 96 | 12.5 |

If >1 class of treatment started on the same day, each class was counted.

Subgroups were defined based on the initial diagnosis of migraine for the patients included in the migraine cohort. It is possible that the same patient may visit multiple facilities in the same month, and the total of the subgroups may not match the patient number of migraine cohort.

*Abbreviations: Anti-CGRP mAbs*, anti-calcitonin gene-related peptide monoclonal antibodies; *CP*, clinic having ≤ 19-bed capacity; *HP*, hospital having ≥ 20-bed capacity; *NSAIDs*, non-steroidal anti-inflammatory drugs.
