# Supplementary material for: Clinical practice for migraine treatment and characteristics of medical facilities and physicians treating migraine: Insights from a retrospective cohort study using a Japanese claims database
Source: PLoS One. 2024 Dec 19;19(12):e0315610. doi: 10.1371/journal.pone.0315610 (PMC11658538; doi:10.1371/journal.pone.0315610)
Supplement: Attachment — Submitted filename: Comments.docx [file pone.0315610.s004.docx]

Dear authors,

This retrospective cohort study aimed to describe real-world clinical practices and treatment patterns in Japanese patients with migraine based on medical facilities and physicians’ specialties. Given that migraine is a major contributor to the global burden of neurological disorders, understanding the relationship between physicians' specialties and the medical environment for migraine, as well as the patterns of migraine treatment across different specialties, would help clarify where the unmet medical needs of migraine patients lie.

Please find my comments for a better presentation of this study.

**Comment 1:**

**Abstract:** In the results section, please mention the F/M ratio and the mean age (SD) of patients.

**Comment 2:**

**Introduction:** This section is well-written and clearly outlines the objective of the study. However, it is better to mention the diagnostic strategy and different types of migraine.

**Comment 3:**

**Results:** The results section aligns with the study's aim; however, it would be beneficial to categorize patients based on migraine type, particularly in the characteristics section. How many patients experienced aura during their first presentation?

**Comment 4:**

**Discussion:** You mentioned that preventive treatments were more commonly used in facilities with specialists. It is important to discuss the significance of this finding and the potential side effects of relying solely on acute treatments. Without access to specialists, patients may resort to using more NSAIDs, which can lead to drug-overuse headaches and other side effects which you have mentioned but please discuss more about the importance of preventive strategy.
